# Leveraging a powerful allogeneic dendritic cell line towards neoantigen-based cancer vaccines

**DOI:** 10.18632/genesandcancer.229

**Published:** 2023-01-30

**Authors:** Dalil Hannani, Estelle Leplus, Karine Laulagnier, Laurence Chaperot, Joël Plumas

**Affiliations:** ^1^PDC*line Pharma, Grenoble, France; ^2^R&D Laboratory, Etablissement Français du Sang Auvergne Rhône-Alpes (EFS AURA), Grenoble, France

**Keywords:** cancer vaccine, neoantigens, plasmacytoid dendritic cells, immunotherapy

## Abstract

In recent years, immunotherapy has finally found its place in the anti-cancer therapeutic arsenal, even becoming standard of care as first line treatment for metastatic forms. The clinical benefit provided by checkpoint blockers such as anti-PD-1/PD-L1 in many cancers revolutionized the field. However, too many patients remain refractory to these treatments due to weak baseline anti-cancer immunity. There is therefore a need to boost the frequency and function of patients’ cytotoxic CD8+ cellular effectors by targeting immunogenic and tumor-restricted antigens, such as neoantigens using an efficient vaccination platform. Dendritic cells (DC) are the most powerful immune cell subset for triggering cellular immune response. However, autologous DC-based vaccines display several limitations, such as the lack of reproducibility and the limited number of cells that can be manufactured. Here we discuss the advantages of a new therapeutic vaccine based on an allogeneic Plasmacytoid DC cell line, which is easy to produce and represents a powerful platform for priming and expanding anti-neoantigen cytotoxic CD8+ T-cells.

## INTRODUCTION

Due to the limited clinical benefit of anti-PD-1/PD-L1 therapy in many cancer indications, there is a renewed interest in therapeutic cancer vaccines to improve clinical responses. Indeed, one of the main explanations for resistance to these immune checkpoint inhibitors (ICI) is the absence of pre-existing anti-tumor immunity or the inadequacy of this immune response [1]. These therapeutic antibodies block the interaction between the inhibitory molecule PD-1 expressed on anti-tumor CD8+ T-cells and its ligand PD-L1, expressed by tumor cells. Their expected *in vivo* mechanism of action is thus to unleash the cytotoxic activity of anti-tumor effectors [2]. In addition, different reports describing the effect of the treatment of patients with ICIs in a neo-adjuvant setting strongly suggested that reinforcing the patient’s own immune system led to the eradication of tumor cells, as evidenced by major or complete pathological responses [3–9]. Therefore, it is becoming increasingly clear that the combination of ICIs with therapeutic cancer vaccines that aimed at priming or enhancing anti-tumor CD8+ T-cell effectors could increase the efficacy of each treatment used separately [10–12].

### Neoantigens as a source of tumor antigens for cancer immunotherapies

Among several potential tumor antigens that can be targeted by the immune system, neoantigens (NeoAgs) appear very attractive because they are tumor cell-specific proteins and unknown to the immune system (i.e., there is no pre-existing central immune tolerance) [13, 14]. NeoAgs were initially described as the result of non-synonymous somatic mutations [14], but they can also be derived from many other genomic abnormalities in the transcriptional and translational process leading to the synthesis of abnormal proteins [15–23]. Interestingly, the frequency of tumor somatic mutations correlates with objective response rates to ICIs in many cancers [24, 25]. Thus, these single nucleotide variants may serve as neoantigens recognized by the immune system, leading to tumor cell death mediated by NeoAg-specific CD8+ T-cells. Very recently, the number and frequency of NeoAg-specific CD8+ T-cells were confirmed to be associated with the clinical outcome of adoptive cell therapy with tumor-infiltrating lymphocytes (TIL) by using an elegant approach [26]. Interestingly, it was also suggested recently that the expansion and activation of NeoAg-specific CD8+ T-cells are associated with the response to ICIs in patients with metastatic urothelial carcinoma [27]. However, despite the considerable number of diverse genomic abnormalities, very few candidates are considered as “good” NeoAgs. This is due to the highly selective molecular machinery allowing the presentation of an immunogenic peptide derived from NeoAgs to the immune system through HLA class I molecules expressed by tumor cells [15, 23]. Recent significant developments in algorithms and deep machine learning have provided opportunities to identify few NeoAgs in the majority of patients, especially in cancers induced by mutagens or DNA mismatch repair [16, 17]. This is probably why therapeutic NeoAg-based cancer vaccines were first developed in melanoma [28–30]. The availability of resected tumors has led to develop vaccines also in glioblastoma, non-small-cell lung cancer (NSCLC), bladder, gastrointestinal, colorectal, urothelial, and pancreatic cancers [31–41]. All studies, except two [36, 37], have so far used private NeoAgs, i.e., identified in a single patient. Most of the clinical studies published are still in phase I or Phase I/II and despite the combination with ICIs, these vaccine approaches are not yet validated clinically.

From an immunological point of view, it is quite surprising that many studies used a vaccine regimen based on local injections of long peptides combined with adjuvants [29, 31–33, 36, 37, 41]. Indeed, these approaches were known to be rather suboptimal to prime and stimulate anti-tumor CD8+ T-cells, and may even generate tolerogenic responses [42–46]. As a result, very weak NeoAg-specific CD8+ T-cell responses have been obtained from patients, in contrast to NeoAg-specific CD4+ T-cells which are not the main effectors of anti-tumor immune response. Indeed, except in rare cases, CD4+ T-cells are not cytotoxic and cannot kill tumor cells due to the lack of expression of HLA class II molecules by tumor cells. RNA-based approaches have also been tested with no significant change in the nature and the amplitude of the anti-tumor response [30, 34]. However, Moderna and Merck have recently reported results on melanoma that will deserve attention when published. The use of adenoviral-based platform has been recently described with some interesting results in few patients [39, 40]. By contrast, the use of mature dendritic cells (DC) loaded with short peptides derived from NeoAgs has demonstrated strong expansions of cytotoxic CD8+ T-cells for many NeoAgs in all melanoma patients tested [28].

### Dendritic cells are essential for the induction of anti-tumor response

Dendritic cells are perfectly equipped to process and present tumor antigen-derived peptides to naive CD8+ T-cells in lymphoid organs, transforming them into effector memory cells capable of reaching to the tumor site and killing tumor cells [47, 48]. They are also very effective in reactivating circulating and tissue-resident anti-tumor memory T-cells [47]. Dendritic cells therefore appear to be of great interest for the development of a cancer vaccine based on NeoAgs, as they directly and efficiently stimulate the appropriate anti-tumor effector cells after injection, avoiding any induction of tolerance [49, 50]. However, to date, given that the main antigen-presenting platforms have used autologous DCs, they have faced major challenges: the cost of manufacturing, reproducibility, feasibility, the availability of sufficient drug product, the suboptimal efficacy of the product, the difficulty of establishing quality control of immune activity, and the heterogeneity of clinical trials since all patients were treated with a different drug product [51]. Except in prostate cancer [52] and very recently in glioblastoma [53], autologous DC-based vaccines have not yet proven their efficiency [54]. Interestingly, numerous issues can be solved using allogeneic dendritic cells [55]. Indeed, allogeneic DCs can be easily manufactured, as the cell source is independent of patients. In addition, the cell drug product is shortly available for the patients when they are enrolled and its potency to stimulate anti-tumor CD8+ T-cells can be checked before infusion.

### Allogeneic plasmacytoid dendritic cells represent an efficient vaccination platform

We have developed a novel approach using an allogeneic plasmacytoid dendritic cell (PDC) line as an antigen-presentation platform showing great potency to prime and expand viral or tumor-specific CD8+ T cells *in vitro* and *in vivo* in a humanized mouse model [55–65]. This off-the-shelf product is scalable, versatile, cost-effective, and guarantees the homogeneity of treatment and clinical results as the same product is used for all patients. This PDC platform, named PDC*vac, was first evaluated with shared tumor-associated antigens in the treatment of melanoma with encouraging results [66]. This first-in-human phase I clinical trial demonstrated PDC*vac safety and biological activity since it primed and expanded anti-tumor CD8+ T-cells in patients. Moreover, we have shown the *in vitro* synergy of PDC*vac with anti-PD-1 drug product leading to the improved expansion of anti-tumor CD8+ T-cells from metastatic melanoma patients. The PDC*vac platform adapted to lung cancer patients (PDC*lung01 product) is currently being evaluated in the treatment of metastatic squamous and non-squamous lung cancer patients in combination with anti-PD-1 antibody (NCT03970746). The preliminary results of this phase I/II are very encouraging in terms of safety, biological, and clinical activities [67].

Given the afore-mentioned advantages of NeoAgs in vaccine approaches, we have exploited the PDC*vac platform in order to activate NeoAg-specific immune response using the same methodology as previously described [58, 66].

**Figure 1:**
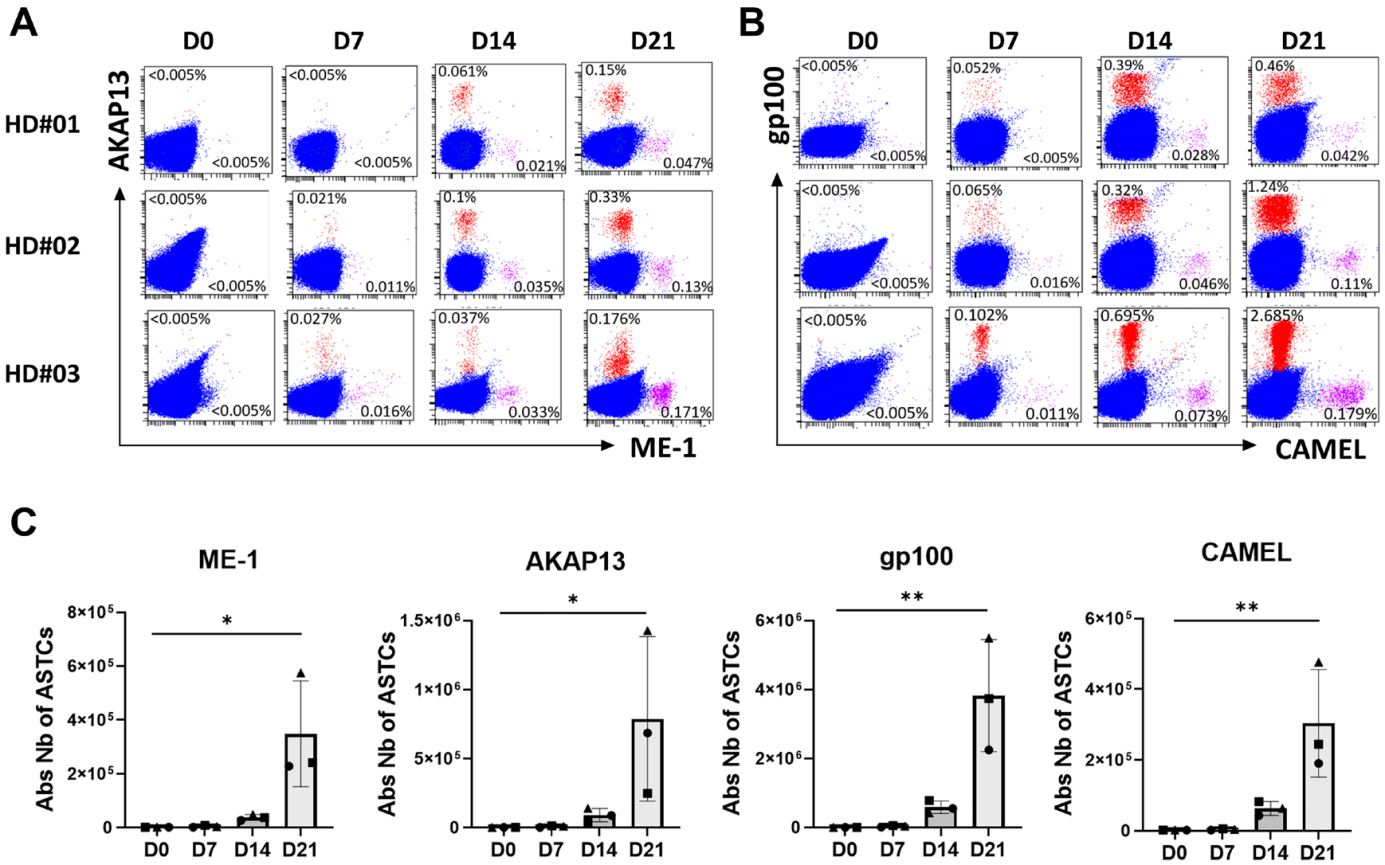
Priming and expansion of NeoAg-specific T-cells by PDC*vac. CD8+ T-cells were purified from the blood of 3 healthy donors (HD#01, HD#02, HD#03) and cocultured with peptide-loaded PDC*line cells during 3 weeks with weekly restimulation at D7 and D14, as detailed in Lenogue et al. [58]. Antigen-specific CD8+ T-cells (ASTC) were measured before (D0) and at different time points during coculture using multimer labeling. The dot plots show the proportion of CD8+ T-cells specific to NeoAg (**A**) and to tumor-associated antigens (**B**) at each time point. At D0, no specific T-cells were detectable above the limit of detection of 0.005%. From D7 to D21, a continuous increase is visible for all antigens. (**C**) The cumulative absolute number of ASTCs is plotted at each time point, for each antigen, and for each of the 3 donors. Each symbol represents a donor: HD#01 is a filled circle, HD#02 a triangle, and HD#03 a filled square. The means of the 3 values +/− SD are shown. One-way Anova statistical analysis was performed. ^*^p < 0.05; ^**^p < 0.01.

We have performed *in vitro* experiments showing that this new product named PDC*neo can effectively prime and expand NeoAg-specific CD8+ T-cells. As a proof of concept, PDC*line cells were loaded with two NeoAgs (ME-1 and AKAP13, Table 1) already described in melanoma and lung cancer patients [28, 68] and two commonly shared tumor-associated antigens as positive controls (gp100, CAMEL). Loaded PDC*line was then cultured with purified healthy donors’ CD8+ T-cells for 3 weeks before detecting specific T-cells with multimer tools (Figure 1). In such experiments, we used CD8+ T-cells purified from healthy donors because they were naive, and thus never encountered NeoAgs. As a consequence, the basal circulating precursor frequencies were expected extremely low (less or equal to 1/1,000,000 in total CD8+ population). However, after weekly stimulations of these rare naive cells with PDC*neo product, a sizeable expansion of antigen-specific CD8+ T-cells was observed as soon as 7 days of co-culture, followed by a powerful expansion at day 21 (Figure 1A and 1B). Indeed, the absolute number of antigen-specific T-cells highly increases from D7 to D21 for both ME-1 and AKAP13. (Figure 1C). As expected, CAMEL- and gp100-specific T-cells were also massively primed and expanded confirming the potency of PDC*line cells (Figure 1C).

**Table 1:** Features of neoantigens

Name	Mutated peptide	Parental Peptide	Reference
**ME-1**	FLDEFME**G**V	FLDEFME**A**V	[68]
**AKAP13 Q285K**	KLMNIQQ**K**L	KLMNIQQ**Q**L	[28]

**Figure 2:**
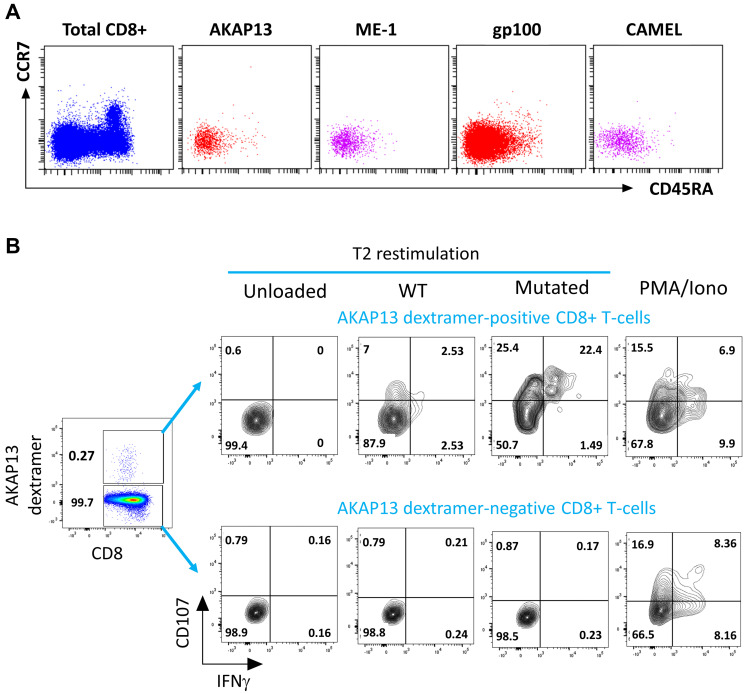
NeoAg-specific T-cells induced by PDC*line cells have an effector/memory phenotype, are functional and specific to the mutated antigen. (**A**) Dot plots showing the CD45RA and CCR7 staining of total CD8+ T-cells and of CD8+ T cells specific to AKAP13, ME-1, gp100, and CAMEL (Donor HD#03). Naive cells are CD45RA^pos^CCR7^pos^ and memory cells are CD45RA^neg^CCR7^neg^. Results are representative of one experiment. (**B**) Illustrative dot plots showing the expression of CD107 and IFNγ by multimer-positive (upper line) and multimer-negative (bottom line) CD8+ T-cells from HD#02 donor upon antigenic stimulation with mutated or wild-type (WT) AKAP13 peptide. Results are representative of two experiments.

Interestingly, after 21 days of culture with PDC*vac, all antigen-specific T-cells displayed an effector/memory phenotype (CCR7^neg^ and CD45RA^neg^; Figure 2A). Moreover, the NeoAg-specific CD8+ T-cells induced by PDC*vac presented functional activity as shown by the expression of CD107 and IFNγ upon stimulation (Figure 2B). Noteworthy, these cells were specific to the mutated form of the neopeptide as they did not react against the wild-type peptide.

Altogether, these data demonstrate that PDC*vac represents an interesting tool for assessing the immunogenicity of neo-epitopes *in vitro*, as well as a powerful vaccine platform for NeoAg-based cancer vaccines. Indeed, PDC*line is a highly potent professional antigen-presenting cell that migrates in lymph nodes and tissues (unpublished data) to directly stimulate peptide-specific CD8+ T-cells. The allogeneic context may bring supplementary activation signal for the immune system. As PDC*line cells are loaded with short peptides, there is no need of antigen transcription, translation, and processing since the peptides are directly loaded on and presented by surface HLA molecules. Finally, the direct presentation of peptides by the dendritic cells themselves avoids any unwanted tolerance induction.

## CONCLUSIONS

**Figure 3:**
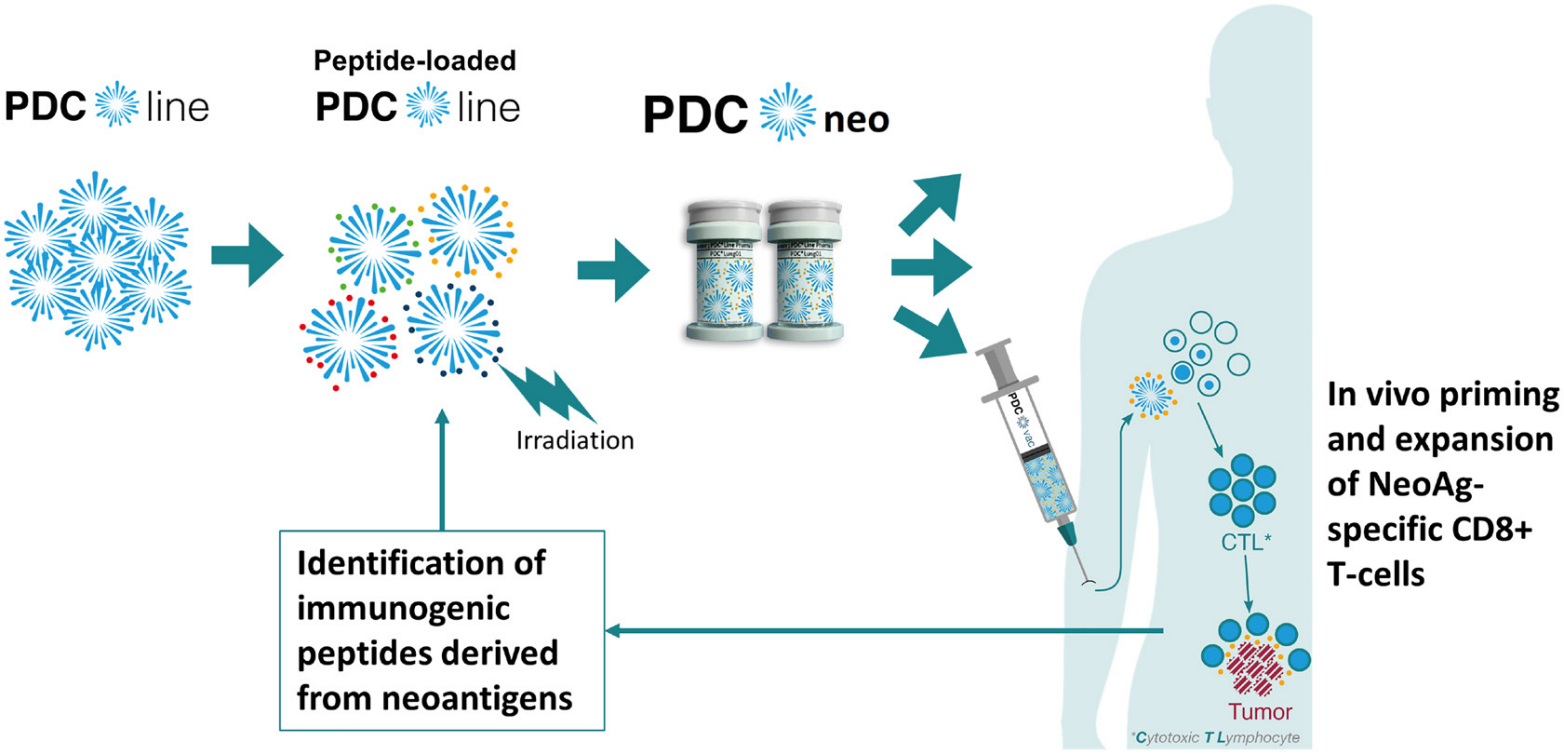
The use of PDC*vac platform to develop NeoAg-based cancer vaccines. Peptides derived from shared or private neoantigens will be loaded on PDC*line cells before their irradiation, packaging, and freezing. The resulting drug product will be thawed on demand and injected into patients to prime and expand NeoAg-specific T-cells *in vivo*, expecting the eradication of tumor cells.

NeoAgs appear attractive candidates to induce specific anti-tumor responses in cancer patients, on top of classical tumor-associated antigens and in association with ICIs. A potent dendritic cell product such as PDC*neo represents a valuable platform to develop NeoAg-based cancer vaccines (Figure 3). We strongly believe that this new delivery technology based on potent PDC*line cells can induce a robust anti-NeoAg CD8+ T-cell immune response for the benefit of patients and could reshape the landscape of NeoAg-based cancer vaccines.
